# Joint image compression and encryption based on sparse Bayesian learning and bit-level 3D Arnold cat maps

**DOI:** 10.1371/journal.pone.0224382

**Published:** 2019-11-18

**Authors:** Xinsheng Li, Taiyong Li, Jiang Wu, Zhilong Xie, Jiayi Shi

**Affiliations:** 1 College of Computer Science, Sichuan University, China; 2 School of Economic Information Engineering, Southwestern University of Finance and Economics, China; Victoria University, AUSTRALIA

## Abstract

Image compression and image encryption are two essential tasks in image processing. The former aims to reduce the cost for storage or transmission of images while the latter aims to change the positions or values of pixels to protect image content. Nowadays, an increasing number of researchers are focusing on the combination of these two tasks. In this paper, we propose a novel joint image compression and encryption approach that integrates a quantum chaotic system, sparse Bayesian learning (SBL) and a bit-level 3D Arnold cat map, so-called QSBLA, for such a purpose. Specifically, the QSBLA consists of 6 stages. First, a quantum chaotic system is employed to generate chaotic sequences for subsequent compression and encryption. Second, as one method of compressive sensing, SBL is used to compress images. Third, an operation of diffusion is performed on the compressed image. Fourth, the compressed and diffused image is transformed into several bit-level cubes. Fifth, 3D Arnold cat maps are used to permute each bit-level cube. Finally, all the bit-level cubes are integrated and transformed into a 2D pixel-level image, resulting in the compressed and encrypted image. Extensive experiments on 8 publicly-accessed images demonstrate that the proposed QSBLA is superior or comparable to some state-of-the-art approaches in terms of several measurement indices, indicating that the QSBLA is promising for joint image compression and encryption.

## 1 Introduction

Images can provide rich information to human vision systems and have become one of the most important ways to transfer information. Currently, thousands of millions of images are produced every day, determining how to efficiently store and transmit such a large number of images is a very challenging task. Due to the bulky data capacity of images, the bytes occupied by images must be reduced to facilitate storage and transmission. The high redundancy and strong correlation that usually exist in an image afford possibilities for image compression. The discrete cosine transform (DCT), discrete Fourier transform (DFT), discrete wavelet transform (DWT), and so on, are widely-used image compression methods [1–3], and some of them have become parts of standards of image compression. Image compression is a trending topic in image processing, and so is image encryption.

Image encryption uses techniques to change the image contents in order to prevent unauthorized people from seeing particularly meaningful contents in the images. Much research has been devoted to image encryption in recent years [4, 5]. Due to the properties of chaotic systems, such as pseudorandomness, unpredictability, ergodicity, and extreme sensitivity to parameters and initial values, chaos-based image encryption has become very popular among image encryption approaches [6, 7]. Individual low-dimensional chaotic systems are easy to crack and hence decrease the security of image encryption. To cope with this issue, possible ways include combining two or more low-dimensional chaotic systems [8, 9], adopting high-dimensional chaotic systems [10], using fractional-order chaotic systems [11, 12], and so on. Recent research has shown that some quantum chaotic systems can achieve good encryption performance, partly because of the extremely sensitive dependence to the initial conditions and/or parameters of quantum chaos [13, 14]. With the chaotic sequences, some schemes such as Latin cubes, S-Box, Arnold cat maps, and so on, can be used to change the positions and/or values of image contents and hence encrypt images [4, 15–17].

Although image compression and image encryption are usually treated as two separate tasks, it is reasonable to combine these two tasks to reduce the image sizes and to prevent the privacy of images from leaking simultaneously. Therefore, it is necessary to study joint image compression and encryption, so-called JICE. In recent decades, JICE has been one of the most acclaimed topics in the field of image processing and information security. Li et al. used a tree structure for JICE in mobile wireless environments [18], while Ou et al. used FPGA to improve a JICE system [19]. To achieve good performance, Yuen et al. integrated a chaotic system, DCT, the Secure Hash Algorithm-1 (SHA-1) and Huffman encoding for JICE, and the experiments confirmed that the presented scheme was efficient for both image compression and encryption [20]. Tong et al. proposed a JICE approach with high security, a good compression effect and high encryption speed by combining DWT and a cross-chaotic map [21]. Li and Lo put forward a JICE scheme based on the JPEG standard, which generated a new orthogonal transform by embedding an additional rotation angle into the 8 × 8 DCT’s flow graph [22]. Zhang and Tong proposed a new joint lossless image encryption and compression approach by combining the integer wavelet transform and set partitioning in hierarchical trees (SPIHT), and the experiments demonstrated that the proposed scheme was able to achieve high security and ideal lossless compression performance [23]. Landir et al. put forward a robust JICE scheme using SPIHT and chaotic maps with noninteger order [24].

Compressive sensing (CS), also referred to as compressive sampling, compressed sensing, or sparse sampling, is an emerging technique in the signal processing community that addresses signal reconstruction by finding sparse solutions to an underdetermined linear system [25–27]. The sparsity of the solutions can be used for compression, so CS has been widely applied for JICE. For example, Zhou et al. used the partial Hadamard matrix controlled by chaotic maps as a measurement matrix for scrambling, and the experimental results demonstrated the validity and reliability of the proposed scheme [28]. The authors also used key-controlled measurement matrix in CS for JICE, and the simulation results demonstrated the scheme was effective and secure [29]. Liu et al. put forward a novel JICE algorithm with fusion for multi-modal images [30]. Zhang et al. proposed a JICE scheme for medical images by CS with a chaos-based Bernoulli measurement matrix and pixel-swapping permutation [31]. A secure and robust JICE algorithm was proposed by integrating DWT, memristive chaotic system, CS and elementary cellular automata (ECA) [32]. Chai et al. used wavelet transformation, zigzag operations, CS and chaos-based measurement matrices to compress and encrypt images [33]. Other JICE approaches with CS and chaotic systems are associated with the Fibonacci-Lucas transform [34], the optimized tensor CS and 3D Lorenz system [35], the 2D CS with a discrete fractional random transform [36], and so on [37–40].

As one type of CS, sparse Bayesian learning (SBL) has shown its superiority in physiological signal analysis [41–43], pattern recognition [44], visual tracking [45] and time series forecasting [46–50] since it was proposed [51, 52]. The existing study has shown that SBL has many advantages over some other compressive sensing models [44, 52, 53]. Regarding image encryption, diffusion and permutation are two typical types of operations that change the values and the positions of pixels in images, respectively [54, 55]. Image compression is naturally an operation of diffusion because it can encode the original images into images with fewer sizes. The Arnold cat map is a popular way to permute the pixels for image encryption [56–59]. As far as the processing unit is concerned, image encryption is usually performed on blocks of pixels, at the pixel level, DNA level (two bits) and bit level. Generally speaking, for a fixed processing power, encryption on lower-level data often involves more pixels to ensure better encryption results can be achieved [15, 60].

Inspired by the extreme sensitivity of quantum chaos, compression ability of CS and permutation power of Arnold, this paper proposes a novel approach that integrates a quantum chaotic system, sparse Bayesian learning and a bit-level 3D Arnold cat map, namely, QSBLA, for joint image compression and encryption. Specifically, the proposed QSBLA consists of 6 stages: 1) a quantum chaotic system is employed to generate a chaotic sequence for subsequent different operations of compression and encryption; 2) SBL is used to compress the original image; 3) a diffusion operation called CDCP is performed on the compressed image; 4) the compressed and diffused image is transformed into a bit-level cuboid, and then, the cuboid is reshaped to one or more bit-level cubes; 5) for each bit-level cube, a 3D Arnold cat map is applied to permute the bits; and 6) all the bit-level cubes are integrated into a bit-level cuboid and then transformed into a pixel-level compressed and encrypted image. The QSBLA is applied to eight publicly accessed test images, and the results indicate that it can achieve good compression performance and has the ability to resist several types of attacks.

The remainder of this paper is structured as follows. Section 2 describes some concepts of the proposed approach. The QSBLA approach is proposed in detail in Section 3. Then, we report and analyze the experimental results in Section 4. Finally, we conclude the paper in Section 5.

## 2 Preliminaries

### 2.1 Quantum chaotic system

A quantum logistic map can be constructed with quantum corrections, as dissipative quantum systems are often coupled to a path of harmonic oscillators [13, 14]. It has been shown that a chaotic map can be created through the very lowest-order quantum corrections by Eq (1) [13].

$$\begin{array}{c} \begin{array}{cc} \phi_{2}\left(x_{n}^{{}^{\prime }}\right) & =r\left(x_{n}^{{}^{\prime }}-{\left|x_{n}^{{}^{\prime }}\right|}^{2}\right)-ry_{n}^{{}^{\prime }},\\ \phi_{2}\left(y_{n}^{{}^{\prime }}\right) & =-y_{n}^{{}^{\prime }}exp\left(-2\beta \right)+exp\left(-\beta \right)r[\left(2-x_{n}^{{}^{\prime }}-x_{n}^{{}^{\prime }*}\right)y_{n}^{{}^{\prime }}-x_{n}^{{}^{\prime }}z_{n}^{{}^{\prime }*}-x_{n}^{{}^{\prime }*}z_{n}^{{}^{\prime }}],\\ \phi_{2}\left(z_{n}^{{}^{\prime }}\right) & =-z_{n}^{{}^{\prime }}exp\left(-2\beta \right)+exp\left(-\beta \right)r[2\left(1-x_{n}^{{}^{\prime }*}\right)z_{n}^{{}^{\prime }}-2x_{n}^{{}^{\prime }}y_{n}^{{}^{\prime }}-x_{n}^{{}^{\prime }}], \end{array} \end{array}$$ (1)

where *x*′ = 〈*α*〉, *y*′ = 〈*δ*α + *δα*〉, *z*′ = 〈*δαδα*〉. *δα* shows a quantum fluctuation about 〈*α*〉, and *β* is a dissipation parameter. Usually, $x_{n}^{{}^{\prime }}$, $y_{n}^{{}^{\prime }}$ and $z_{n}^{{}^{\prime }}$ are complex numbers with $x_{n}^{{}^{\prime }*}$ of the complex conjugate of $x_{n}^{{}^{\prime }}$, similarly for $z_{n}^{{}^{\prime }}$. After setting *r* and *β*, we can iterate Eq (1) with initial parameters $x_{0},y_{0},z_{0},x_{0}^{*}$ and $z_{0}^{*}$ to produce a chaos sequence. In the experiment, once *x*_0_, *y*_0_ and *z*_0_ are set to real numbers, $x_{0}^{*}$ and $z_{0}^{*}$ are the same as *x*_0_ and *z*_0_, and then they will be replaced by *x*_0_ and *z*_0_.

$$\begin{array}{c} \begin{array}{cc} x_{n+1}^{{}^{\prime }} & =\left(1-\epsilon \right)\phi \left(x_{n}^{{}^{\prime }}\right)+\epsilon \phi \left(y_{n}^{{}^{\prime }}\right),\\ y_{n+1}^{{}^{\prime }} & =\left(1-\epsilon \right)\phi \left(y_{n}^{{}^{\prime }}\right)+\epsilon \phi \left(z_{n}^{{}^{\prime }}\right),\\ z_{n+1}^{{}^{\prime }} & =\left(1-\epsilon \right)\phi \left(z_{n}^{{}^{\prime }}\right)+\epsilon \phi \left(x_{n}^{{}^{\prime }}\right). \end{array} \end{array}$$ (2)

Iterating Eq (2) with real initial parameters *x*_0_, *y*_0_, *z*_0_ and the real constant *ϵ*, the chaos sequence of real numbers is produced.

### 2.2 Arnold cat map

Arnold’s cat map is one of the well-known chaotic maps, and the name was from the fact that Vladimir Arnold demonstrated its effects on an image of a cat in the 1960s [61, 62]. When applying the map to encrypt an image, the image appears to be randomly permuted. However, if the map is repeated a certain number of times, the original image will appear again. The 2D Arnold cat map, formulated as Eq (3), has been widely used in image encryption.

$$\begin{array}{c} {}[\begin{array}{c} x^{{}^{\prime }}\\ y^{{}^{\prime }} \end{array}]=[\begin{array}{cc} 1 & a\\ b & ab+1 \end{array}]*[\begin{array}{c} x\\ y \end{array}]mod\;N, \end{array}$$ (3)

where *a* and *b* are two specified positive integers, (*x*′, *y*′) is the new position of the original image position (*x*, *y*), and *N* is the size of the original square image. By adding two extra parameters *c* and *d*, we can obtain a 3D Arnold cat map, as defined by Eq (4):

$$\begin{array}{c} {}[\begin{array}{c} x^{{}^{\prime }}\\ y^{{}^{\prime }}\\ z^{{}^{\prime }} \end{array}]=[\begin{array}{ccc} 1 & 0 & a\\ bc & 1 & abc+c\\ bcd+b & d & abcd+ab+cd+1 \end{array}]*[\begin{array}{c} x\\ y\\ z \end{array}]mod\;N, \end{array}$$ (4)

where *a*, *b*, *c* and *d* are four specified positive integers, *N* is the size of the cubic image, and *x*, *y*, and *z* represent the original positions in the direction of height, width and depth, respectively. Likewise, *x*′, *y*′ and *z*′ stand for the new positions in the direction of height, width and depth, respectively.

Accordingly, the inverse transformation of Eq (4) can be formulated as Eq (5).

$$\begin{array}{c} {}[\begin{array}{c} x\\ y\\ z \end{array}]=[\begin{array}{ccc} 1+ab & ad & -a\\ 0 & 1+cd & -c\\ -b & -d & 1 \end{array}]*[\begin{array}{c} x^{{}^{\prime }}\\ y^{{}^{\prime }}\\ z^{{}^{\prime }} \end{array}]mod\;N. \end{array}$$ (5)

## 3 QSBLA: The proposed joint image compression and encryption approach

### 3.1 Quantum chaotic sequence generation

**Fig 1:**
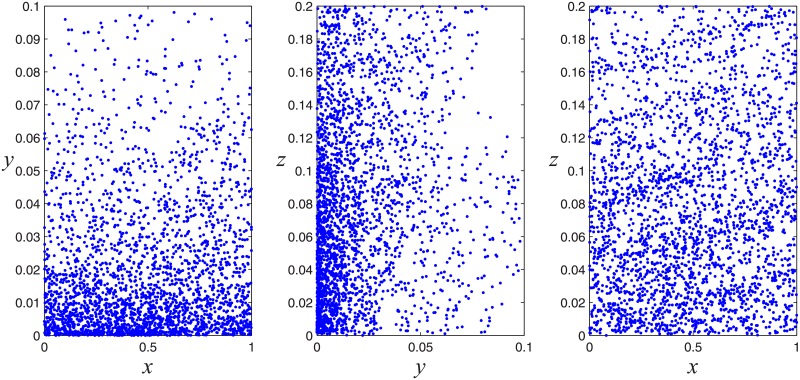
Chaotic attractor of the quantum chaotic system.

The quantum chaotic sequence of QSBLA is generated from Eqs (1) and (2). When the parameters (*ϵ*, *r*, *β*) = (0.001, 8, 3.32) and initial values (*x*_0_, *y*_0_, *z*_0_) = (0.4239, 0.0239, 0.0239) for Eqs (1) and (2), the attractors of the quantum chaotic system are shown in Fig 1. *r* is a very important parameter that affects the attractors’ distribution area in the quantum chaotic system significantly, which can be lines or random points. Fig 1 shows that the map has good chaotic characteristics and randomness. In quantum chaotic theory, chaos cannot be measured in the same way as defined in classical dynamics (i.e., through Lyapunov exponents) because the evolution operator in quantum mechanics is coupled. Here, Fig 1 verifies the chaotic property of the quantum system by the random distribution of the points.

Then, sequence *K* is composed of $x_{n}^{{}^{\prime }}$, $y_{n}^{{}^{\prime }}$, $z_{n}^{{}^{\prime }}$, formulated as Eq (6).

$$\begin{array}{c} K=[\left(x_{1}^{{}^{\prime }},y_{1}^{{}^{\prime }},z_{1}^{{}^{\prime }}\right),\left(x_{2}^{{}^{\prime }},y_{2}^{{}^{\prime }},z_{2}^{{}^{\prime }}\right),\cdots ,\left(x_{n}^{{}^{\prime }},y_{n}^{{}^{\prime }},z_{n}^{{}^{\prime }}\right),\cdots ]. \end{array}$$ (6)

The purposes of the sequence *K* for JICE are twofold: (1) sorting the subsequence of *K* to obtain the index of the original data for permutation and diffusion; and (2) using the subsequence of *K* to form the measurement matrix for SBL. In the proposed approach, the *i*-th point in *K* is mapped to the integral range of [0, 255] by Eq (7) for both purposes.

$$\begin{array}{c} k_{i}=mod\left(\lfloor mod\left(\left(|k_{i}|-\lfloor |k_{i}|\rfloor \right)\times 10^{15}\right),10^{8})\rfloor ,256\right), \end{array}$$ (7)

where *mod* and |⋅| are the modulo and the absolute value operation respectively, and ⌊⋅⌋ denotes the flooring operation [12]. The discretization through Eq (7) can improve the randomness of *K* to enhance the encryption.

### 3.2 Sparse bayesian learning

Sparse Bayesian learning (SBL) was first proposed for regression and classification by Tipping in 2001, and its main idea is to obtain sufficiently sparse solutions from the data in a mapped high-dimensional space via kernel-tricks by a Bayesian framework [51]. Later, the nonkernel SBL was proposed and widely applied to signal recovery, compressive sensing and sparse representation [41, 52].

From the perspective of compressive sensing, if the sparsity exists in the original signal or in its corresponding transform domains, the high-dimensional signal has the potential to be projected into a low-dimensional space by a dictionary (also known as a measurement matrix) to achieve compression [32]. Moreover, the original signal can then be recovered as close as possible via the compressed signal.

Mathematically, sparse signal recovery can be defined by Eq (8):

$$\begin{array}{c} y=\Theta x+\epsilon , \end{array}$$ (8)

where Θ ∈ *R*^*P*×*Q*^ is a measurement matrix with *P* samples and each has *Q* features, and *y* = [*y*_1_, *y*_2_, ⋯, *y*_*P*_]^*T*^ is a target vector, *ϵ* is noise and *x* = [*x*_1_, *x*_2_, ⋯, *x*_*Q*_]^*T*^ is the vector to be resolved to quantify the weights of the columns in Φ. SBL aims to seek a vector *x* that has as many zero entities as possible while it still approximates the targets *y* accurately [48, 52].

With an approximative zero-mean Gaussian noise (*ϵ* in Eq (8)) with unknown variance *σ*^2^, the framework of SBL assumes the Gaussian likelihood model as Eq (9):

$$\begin{array}{c} p\left(y|x;\sigma^{2}\right)={\left(2\pi \sigma^{2}\right)}^{-\frac{P}{2}}exp\left(-\frac{1}{2\sigma^{2}}||y-{\Phi w||}^{2}\right). \end{array}$$ (9)

Now, the task of seeking maximum likelihood estimates for *x* is transformed into the task of seeking the minimum *ℓ*_2_-norm solution to Eq (8). However, it can usually find nonsparse solutions. To cope with this issue, the SBL estimates a parameterized prior, instead of using a fixed prior as adopted in some other compressive sensing approaches, over weights from the data by Eq (10):

$$\begin{array}{c} p\left(x;\gamma \right)=\prod_{i=1}^{Q}{\left(2\pi \gamma_{i}\right)}^{-\frac{1}{2}}exp(-\frac{x_{i}^{2}}{2\gamma_{i}}), \end{array}$$ (10)

where *γ* = [*γ*_1_, *γ*_2_, ⋯, *γ*_*Q*_]^*T*^ is a vector to control the prior variance of each weight, and *Q* hyperparameters need to be estimated in total. There are two key steps to estimate these hyperparameters, i.e., marginalizing over the weights as well as performing the maximum likelihood optimization algorithm.

When compared with other CS algorithms, SBL has the following advantages: 1) The recovery performance is robust to the characteristics of the measurement matrix; 2) SBL usually outperforms some other CS algorithm regarding local and global convergence; 3) The solutions of SBL are sparser than those of LASSO-type algorithms; and 4) Some robust learning rules can be used to automatically estimate the regularization term of SBL to achieve good results of compressive sensing. Therefore, SBL is suitable for image compression [44, 53]. For more details, interested readers can refer to [52].

### 3.3 SBL-based image compression

The traditional image lossy compression methods, such as FT, DCT, DWT and so on, have been successfully applied to some compression standards such as JPEG, MPEG, H.26X and so on. With ever-increasing applications of these methods, some of their disadvantages have emerged. For example, when the compression ratio (CR) is very high, the decompression image will have such extensive obvious rectangle block shapes that difficulties in introducing human visual characteristics arise. Therefore, improving the quality of decompression images, increasing the CR and speeding up the encoding and decoding procedure are consistent directions followed by researchers.

CS can efficiently reconstruct a signal from very few samples by finding solutions to underdetermined linear systems.

This signal technique fully exploits the sparsity of a signal to compress information when receiving.

For a given sparse signal *x* of length *N*, it can be expressed as Eq (11):

$$\begin{array}{c} x=\Psi s, \end{array}$$ (11)

where *s* is a transform coefficient vector of length *M* (*M* ≪ *N*), and Ψ is an orthogonal transform matrix, also known as a sparse basis matrix.

Assuming *ϵ* = 0, Eq (8) is deduced to as follows

$$\begin{array}{c} y=\Phi x=\Phi \Psi s=\Theta s, \end{array}$$ (12)

where Φ is called the measurement matrix as in Section 3.2. In the scheme, sparse signals *s* represent the plain image *I*. The sparse representation of original signals *s* by Ψ is executed by DCT. The measurement matrix Φ is formed by the random integers from the quantum chaotic sequence *K*, and Φ is rescaled within [−0.5, 0.5] by the following Eq (13).

$$\begin{array}{c} \Phi_{rh\times h}⇐\frac{K-min(K)}{max(K)-min(K)}-0.5, \end{array}$$ (13)

Thus, for a pixel-level image *I* with size of *h* × *w* and different compression ratios *CR* = *r*, 0 < *r* < 1, we choose the top *r* × *h* rows from the image after the sparse representation result to compress it. Then, the size of Ψ is *h* × *h*, and the size of Φ is *rh* × *h*.

After the transformation of *I* with ΦΨ, the data type of *y* is double. The *y* has to be stored as an unsigned 8-bit integer. Here, we need to map *y* within [0, 255] and record the rounded result as integers by Eq (14). By doing so, we can save 50% of the storage space for the encrypted image and one pixel only needs a one-byte space. Therefore, the maximum and minimum of *y* need to be stored as keys.

$$\begin{array}{c} y⇐\lfloor \frac{y-min(y)}{max(y)-min(y)}\times 255\rfloor . \end{array}$$ (14)

Finally, the steps of SBL compression are the following.

Step 1: Generate a sparse representation matrix Ψ.
Step 2: Generate a measurement matrix Φ and scale it by Eq (13).
Step 3: Generate a compressed image *y* by Eq (12) and map *y* to [0, 255] by Eq (14).

In contrast, the steps of SBL decompression are listed below:

Step 1: Rescale *y* to the real numbers within the original minimum and maximum.
Step 2: Generate the measurement matrix Φ in the same way from *K* and scale it within [−0.5, 0.5].
Step 3: Likewise, generate the sparse transformation matrix Ψ with DCT.
Step 4: Compose Θ = ΦΨ.
Step 5: Learn *μ* through *y* and Θ by SBL.
Step 6: Recover $\hat{I}=\hat{s}=\Psi^{T}\mu$. Here, $\hat{I}$ is the reconstructed lossy image.

Moreover, the pseudo code of SBL compression and decompression is demonstrated in Algorithm 1 and Algorithm 2 which provide more details of SBL-based image compression.

### 3.4 Bit-level 3D Arnold cat map

Since the 3D Arnold cat map transformation can be conducted on cubes only, an image has to be reshaped to one or several cubes before encryption. For a pixel-level image with a size of *h* × *w*, where *h* and *w* indicate the height and width of the image, respectively, we first transform it into a bit-level cubic with a size of *h* × *w* × 8. Then, we transform the bit-level cubic into one or more cubes using a previously proposed I2C algorithm [15]. For example, a pixel-level image with size 512 × 512 can be transformed into a bit-level cubic with a size 512 × 512 × 8, and then it can be further transformed into a bit-level cube with size 128 × 128 × 128. Similarly, a 256 × 256 pixel-level image can be transformed into 2 64 × 64 × 64 bit-level cubes.

**Fig 2:**
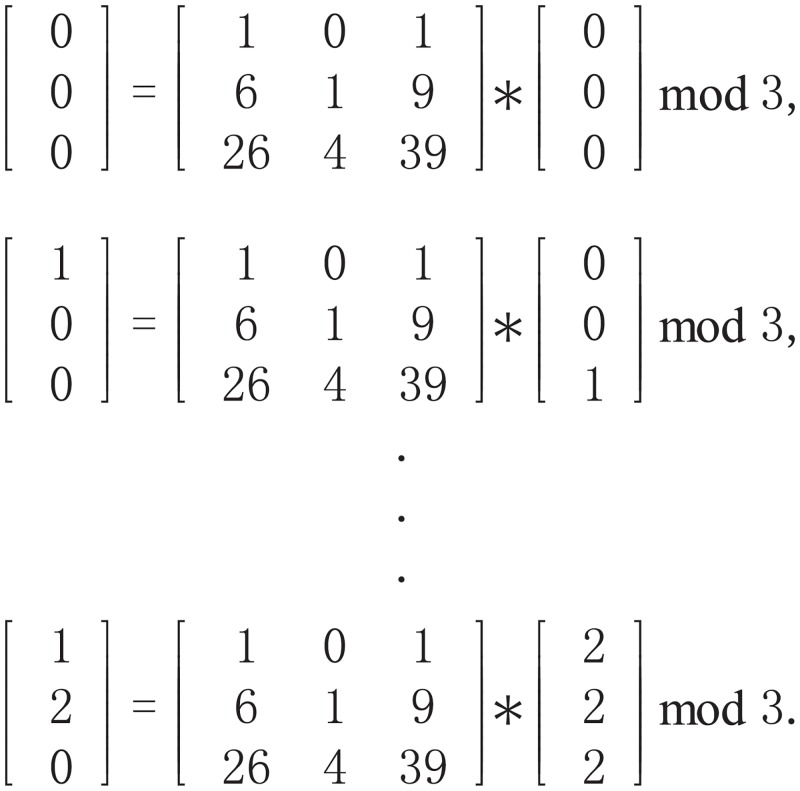
An example of the 3D Arnold cat map.

Once we obtain the cube(s), bit-level permutation can be conducted via the 3D Arnold cat map defined by Eq (4). For example, given a 3 × 3 × 3 input cube and parameters (*a*, *b*, *c*, *d*) = (1, 2, 3, 4), the original 27 positions are 000, 001, 002, ⋯, 222. With the 3D Arnold cat map permutation, we can obtain new values for the 27 positions by (0,0,0) → (0,0,0), (0,0,1) → (1,0,0), ⋯, (2,2,2) → (1,2,0), i.e., the value on the left side of the arrow is moved to the corresponding right side, as shown in Fig 2:

### 3.5 QSBLA: The proposed joint image compression and encryption approach

**Fig 3:**
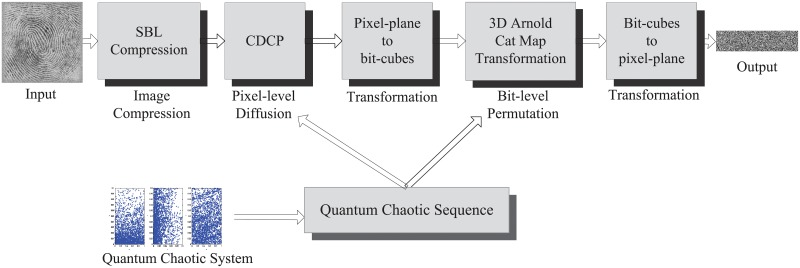
The JICE flowchart of QSBLA.

The flowchart of the QSBLA is shown in Fig 3. After generating the quantum chaotic sequence, the JICE procedure consists of five steps: 1) SBL compression, 2) ciphertext diffusion in the crisscross pattern (CDCP) diffusion [54], 3) transformation from a 2D pixel-level image to bit-level cubes [15], 4) 3D Arnold cat map permutation, and 5) transformation from bit-level cubes to a 2D pixel-level image. The diffusion aims to change the pixel values while the permutation aims to rearrange the positions of bits.

The procedure of the proposed QSBLA is described in detail as follows:

Step 1: Generating the quantum chaotic sequence: Generate the quantum chaotic sequence with initial keys by Eqs (1) and (2).
Step 2: SBL compression: Compress the plain image through Eq (8) detailed in Section 3.3. The DCT transformation is used for sparse representation. Measurement matrix Φ is composed of the chaotic sequence *K*.
Step 3: CDCP diffusion: Conduct CDCP operations on the compressed image following the steps in [54].
Step 4: Transformation from a 2D pixel-level image to bit-level cubes: Use the I2C algorithm proposed in [15] to transform the 2D pixel-level compressed and diffused image to several bit-level cubes for the convenience of the subsequent 3D Arnold cat map.
Step 5: 3D Arnold cat map permutation: For each bit-level cube, the 3D Arnold cat map is used to permute the bits as in Section 3.4. Since the permutation on bits can result in the change of corresponding pixel values, such an operation also has the effect of diffusion.
Step 6: Transformation from bit-level cubes to a 2D pixel-level image: All the bit-level cubes are first merged into a bit-level cuboid, and then the cuboid is transformed to a pixel-level 2D image, i.e., the compressed and encrypted image.

The decryption and decompression is the inverse procedure of the compression and encryption. In addition, the pseudo codes of the QSBLA and the corresponding decryption and decompression procedures are listed in detail in Algorithm 1 and Algorithm 2, respectively.

**Algorithm 1** QSBLA Compression and Encryption

**Input:** *K* (Chaos sequence), *I* (Plain image), *CR* (Compression Rate)

**Output:** *C* (The compressed and encrypted image)

1: **Begin:**

2: **//SBL Compression**

3: [*h*, *w*] ← size(*I*)

4: *N* ← *CR* × *h*

5: *ψ* ← Generate the DCT transform matrix of size (*w*, *h*)

6: *α* ← *ψ* × *I*

7: *ϕ* ← *K* by Eq (13)

8: *y*_1_ ← *ϕ* × *ψ* × *α* by Eq (12)

9: *y*_2_ ← Scale *y*_1_ by Eq (14)

10: **//Encryption**

11: *cd* ← Conduct CDCP on *y*_2_ as in [54]

12: *b* ← Perform bit XOR on *cd* with *K*

13: *bm* ← Transform pixel *b* to a bit matrix as in [15]

14: *cu* ← Divide bit matrix *bm* to cubes as in [15]

15: *mp* ← Permutate cubes *cu* by 3D Arnold cat map with *K* as in Section 3.4

16: *C* ← Transform *mp* to a pixel image

17: **return** *C*

18: **End**

**Algorithm 2** QSBLA Decryption and Decompression

**Input:** *K* (Chaos sequence), *C* (Compressed and encrypted image), *CR* (Compression Rate), *y*_*max*_/*y*_*min*_ (Maximum/minimum of *y*_1_ in Algorithm 1)

**OutPut**: $\hat{I}\left(Recovered\;image\right)$

1: **Begin:**

2: **//Decryption**

3: *bm* ← Transform the compressed and encrypted pixel image *C* to a bit matrix as in [15]

4: *cu*← Divide the bit matrix *bm* to cubes as in [15]

5: *mp*← Reversely permutate cubes *cu* by 3D Arnold cat map with *K* as in Section 3.4

6: *I*_*a*_ ← Transform *mp* to a pixel image

7: *b*← Perform bit XOR on *I*_*a*_ with *K*

8: *I*_*c*_ ← Reversely conduct CDCP on *b* as in [54]

9: **//SBL Decompression**

10: [*N*, *w*] ← size(*Ic*)

11: *h* ← *N*/*CR*

12: *ψ* ← Generate the DCT transform matrix of size (*w*, *h*)

13: *ϕ* ← *K* by Eq (13)

14: *y*_1_ ← Reversely scale *I*_*c*_, *y*_*max*_, and *y*_*min*_ by the inverse function of Eq (14)

15: *θ* ← *ϕ* × *ψ*

16: *μ*← Recover signal from *y*_1_ and *θ* as in Section 3.4

17: $\hat{I}\leftarrow \psi^{T}\times \mu$

18: **return** $\hat{I}$

19: **End**

## 4 Experimental results

### 4.1 Experimental settings

To measure the performance of the QSBLA, some state-of-the-art image encryption approaches, such as CDCP [54], the hyperchaotic and DNA sequence-based method (HC-DNA) [63], a class hyperchaos-based scheme (CHC) [55], and an image cipher scheme with block-based scrambling and image filtering (IC-BSIF) [64], are tested with some popular evaluation indices. We also compare with some popular compression and encryption schemes for some specific images with some evaluation indices. The parameters for the above schemes are set as those in the corresponding papers. The parameters of the QSBLA are described as the following. The initial parameters for the quantum chaotic system are *x*_0_ = 0.4239, *y*_0_ = 0.0239 and *z*_0_ = 0.0239. Additionally, the other parameters are *ϵ* = 0.001, *r* = 3.99 and *β* = 6.

The original integral chaotic sequence generated by the quantum chaotic equations is adopted from the point at which it begins, that is to say, the start position of the chaotic sequence is 1.

The round of the Arnold transformation is also set to 1. If necessary, the start position of the sequence and the rounds of the Arnold cat map can be also used as the security keys.

To demonstrate the performance of the proposed QSBLA, we use 8 images for experiments. The names and sizes of the testing images are listed in Table 1. All the images are publicly-accessed and are very popular in the literature on image processing [65–67].

**Table 1:** Testing images.

Image	Size (*w* × *h*)	Image	Size (*w* × *h*)
Finger	256 × 256	Cameraman	256 × 256
Barbara	512 × 512	Airfield	512 × 512
Baboon	512 × 512	Peppers	512 × 512
Texture	512 × 512	Boats	512 × 512

In the experiments, we use the structural similarity (SSIM) and the peak signal-to-noise ratio (PSNR) for compression effect analysis, the key space and sensitivity for security key analysis, histogram, information entropy and correlation for statistical analysis, the number of pixels change rate (NPCR) and the unified average changing intensity (UACI) for differential attack analysis, and noise and data loss for robustness analysis. Furthermore, we analyze known-plaintext and chosen-plaintext attacks, as well as computation time.

All the experiments were conducted with MATLAB 2016b on a 64-bit Windows 7 with 8 GB memory and an i3 CPU at 3.4 GHz.

### 4.2 Encrypted images and decrypted images

**Fig 4:**
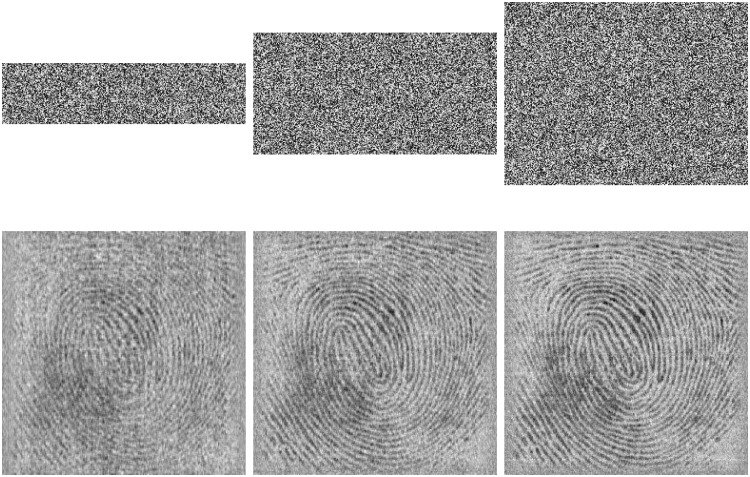
The encryption images of Finger and their recovered images with different CRs of 0.25, 0.5 and 0.75. The first row shows their compressed and encrypted images by QSBLA while the second row shows their corresponding recovered images.

**Fig 5:**
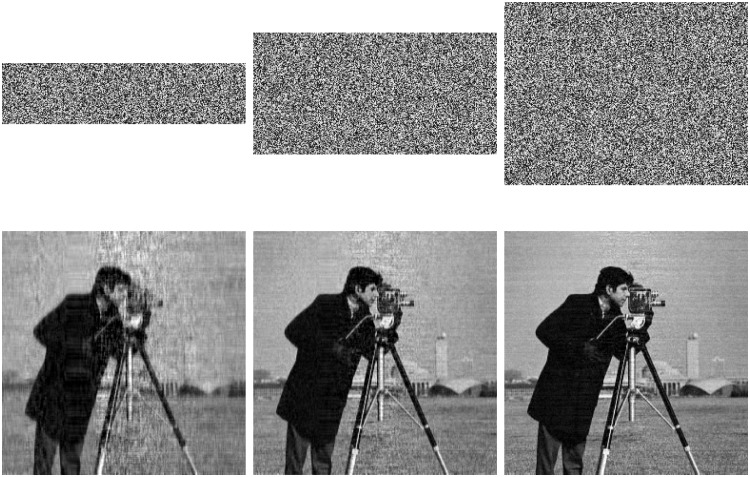
The encryption images of Cameraman and their recovered images with different CRs of 0.25, 0.5 and 0.75. The first row shows their compressed and encrypted images by QSBLA while the second row shows their corresponding recovered images.

**Fig 6:**
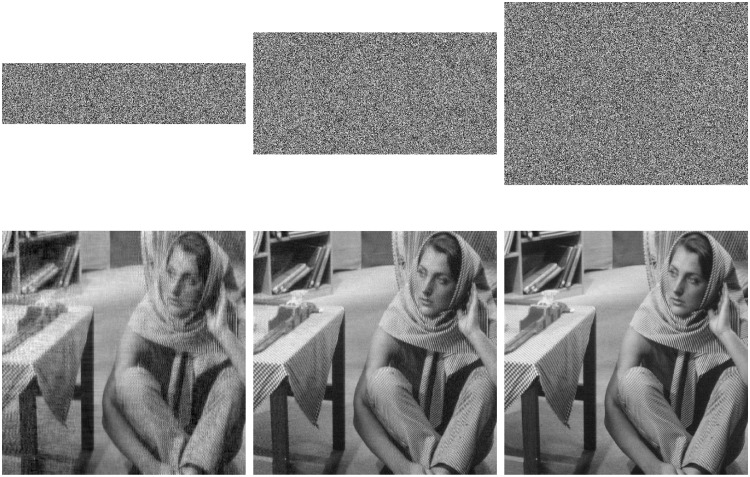
The encryption images of Barbara and their recovered images with different CRs of 0.25, 0.5 and 0.75. The first row shows their compressed and encrypted images by QSBLA while the second row shows their corresponding recovered images.

With the QSBLA, the encryption images of Finger, Cameraman and Barbara, and their corresponding recovered images are shown in Figs 4–6, respectively. From the first column to the third column, their CRs are 0.25, 0.5 and 0.75. The heights of compressed and encrypted images are 1/4, 1/2 and 3/4 of their corresponding original images, as shown in the first row. The second row shows the corresponding recovered images from the first row.

As can be seen from Figs 4–6, when the CR increases, the recovered images gradually achieve better quality. When *CR* = 0.75, it is hard to find out the difference between the plain images and the corresponding recovered images based on human vision.

When we compare the results of Cameraman by QSBLA and those by Ref. [29], we can find that the pixels in compressed and encrypted image by QSBLA distribute more uniformly than those by Ref. [29]. At the same time, the quality of the recovered Cameraman by QSBLA is better than that by Ref. [29]. Similar results can be found from the results of Barbara by QSBLA and those by Ref. [32].

### 4.3 The effect of the compression

#### 4.3.1 Structural similarity (SSIM)

The structural similarity index (SSIM) defined with Eq (15) is used to measure the quality of the decompression and decryption image [60].

$$\begin{array}{c} SSIM\left(x,y\right)=\frac{\left(2\mu_{x}\mu_{y}+C_{1}\right)\left(2\sigma_{xy}+C_{2}\right)}{\left(\mu_{x}^{2}+\mu_{y}^{2}+C_{1}\right)\left(\sigma_{x}^{2}+\sigma_{y}^{2}+C_{2}\right)}, \end{array}$$ (15)

where *x* and *y* are two images, *μ*_*x*_ and *μ*_*y*_ are the average values of *x* and *y*, $\sigma_{x}^{2}$ and $\sigma_{y}^{2}$ are the variance of *x* and *y*, respectively, and *σ*_*xy*_ is the covariance of *x* and *y*. In addition, *C*_1_ = (0.01 × *L*)^2^, *C*_2_ = (0.03 × *L*)^2^, where *L* = 255 is the gray level of the pixel value. The smaller the SSIM is, the greater the difference of both images and the less the similarity.

The SSIM values of the testing images by the proposed QSBLA are shown in Table 2. From this table, with the compression ratio increasing, the variation of SSIM are consistent with the compression data storage, showing that lower CR results in lower quality of the recovered image. Although the values of SSIM in Table 2 are all less than 0.9, the decompression and decryption images in Figs 4 to 6 appear very similar to the results in Ref. [29] and Ref. [32], whose mean SSIM (MSSIM) is greater than 0.96.

**Table 2:** The SSIM values of different images under different compression ratios.

Image	Finger	Cameraman	Barbara	Airfield	Baboon	Peppers	Texture	Boats
CR = 0.25	0.5044	0.5237	0.5993	0.5107	0.4662	0.6501	0.5749	0.6955
CR = 0.50	0.7419	0.6882	0.7979	0.7162	0.6581	0.7826	0.7721	0.8634
CR = 0.75	0.8838	0.7580	0.8822	0.8334	0.8045	0.8368	0.8966	0.8908

#### 4.3.2 Peak signal-to-noise ratio (PSNR)

The peak signal-to-noise ratio (PSNR) is a widely used index to quantify the similarity between the plain image and the recovered image after processing to judge the effectiveness of compression, which is computed by Eqs (16) and (17) [60].

$$\begin{array}{c} \text{MSE}=\frac{1}{N}\sum_{i=1}^{h}\sum_{j=1}^{w}{\left(I(i,j)-E(i,j)\right)}^{2}, \end{array}$$ (16)

$$\begin{array}{c} \text{PSNR}=10\times \log_{10}\left(\frac{255\times 255}{MSE}\right), \end{array}$$ (17)

where *I*(*i*, *j*) and *E*(*i*, *j*) are the pixel gray values of the plain image and the recovered image, respectively, the MSE is the mean variance of the plain image and the recovered image, *N* = *w* × *h* is the pixel number of the image, and *w* and *h* is the width and height of the image, respectively.

The PSNR of the QSBLA is compared with the methods of Ref. [29] and Ref. [32], and the results are listed in Table 3. From Table 3, it can be seen that the PSNR by the QSBLA with the image Cameraman increases steadily as the *CR* varies from 0.25 to 0.75. Although the PSNR values of Cameraman by QSBLA are not as good as those of Ref. [29] and Ref. [32], they are very close to the best results, showing that the proposed QSBLA is comparable to the competitive approaches in terms of the compression effect.

**Table 3:** The compression performance PSNR of different algorithms.

Image	CR	QSBLA	Ref. [29]	Ref. [32]
Cameraman	CR = 0.25	22.22	22.64	**25.23**
	CR = 0.5	26.65	26.71	**29.43**
	CR = 0.75	29.80	**30.85**	28.93

### 4.4 Security key analysis

An effective encryption scheme should be extremely sensitive to any small changes in its security key and has an enough large key space. Therefore, key space and sensitivity to the security key are two important factors in image encryption. Both a large key space and extreme sensitivity is very helpful to resist any brute-force attacks.

#### 4.4.1 Key space

The security keys of the proposed QSBLA include 6 values to generate the chaotic sequence, i.e., $\left(x_{0}^{0},y_{0}^{0},z_{0}^{0},r,\beta ,∊\right)$, as well as the maximum and minimum of *y*_1_ in Algorithm 1, i.e., (*y*_*max*_, *y*_*min*_). That is to say, the security keys are composed of 8 values, i.e., $\left(x_{0}^{0},y_{0}^{0},z_{0}^{0},r,\beta ,∊,y_{max},y_{min}\right)$. If each initial value has a precision of 10^−15^, the size of the key space is 10^15 × 8^ = 10^120^ ≈ 2^399^. From the view of cryptology, the key space whose size is larger than 2^100^ implies high-level security [68, 69]. Therefore, the key space of the proposed QSBLA is so large that it can resist all kinds of brute-force attacks. Moreover, the start position and rounds of the chaotic sequence to form the measurement matrix of CS or the Arnold transform matrix can also be used as security keys to further expand the key space of the QSBLA.

#### 4.4.2 Sensitivity to the security key

An image encryption algorithm with an extreme sensitivity requires that any tiny changes in the keys will produce a completely different cipher image, that is to say, if the security key changes slightly, the recovered image will become totally different from the plain image.

**Fig 7:**
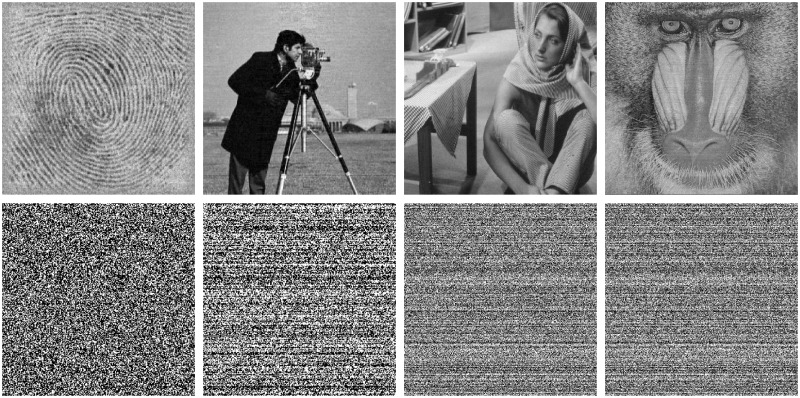
Decrypted decompression images of *CR* = 0.75 with the right key (1st row) and the wrong key (2nd row). Only 10^−15^ is added to *x*_0_ of the right key to form the wrong key.

To verify the sensitivity to the security key of the QSBLA, we decrypt the encrypted images twice but with slightly different keys to result in two encrypted images. First, the encryption keys ($x_{0}^{0}=0.4239$, $y_{0}^{0}=0.0239$, $z_{0}^{0}=0.0239$, *r*^0^ = 3.99, *β*^0^ = 6, *ϵ*^0^ = 0.001, *y*_*max*_, *y*_*min*_) are used to decrypt the encrypted images, where *y*_*max*_ and *y*_*min*_ are associated with the contents of the corresponding plaintext image. Then, the encrypted compression images are decrypted with slightly different keys $(x_{0}^{1}=0.4239+10^{-15}$, $y_{0}^{1}=0.0239$, $z_{0}^{1}=0.0239$, *r*^1^ = 3.99, *β*^1^ = 6, *ϵ*^1^ = 0.001, *y*_*max*_, *y*_*min*_). The experimental results of Finger, Cameraman, Barbara and Baboon are shown in Fig 7. As seen, even a very slight change of 10^−15^ with the correct keys results in completely different recovered images from the decrypted images. The wrong decrypted decompression images resemble a random number map and show no visual information about the plain images. It validates that the QSBLA is extremely sensitive to the security key.

### 4.5 Statistical analysis

For the purpose of performance evaluation of the QSBLA, some typical statistical analysis, such as histogram analysis, information entropy and correlation analysis are adopted in the experiments.

#### 4.5.1 Histogram analysis

Histograms are popular and effective ways to measure the distribution of all the pixel values in an image. In general, the histogram of a plain image is unevenly distributed, while that of an encrypted image produced by a good encryption method should have a uniform distribution. A uniform distribution of a histogram always represents a totally random-like image with relatively low correlations among neighborhood pixels and has the least probability of hacking the encrypted image to recover the corresponding plain image. In other words, the more even the histogram of the encrypted image is, the better the encryption scheme is in resisting histogram attacks.

**Fig 8:**
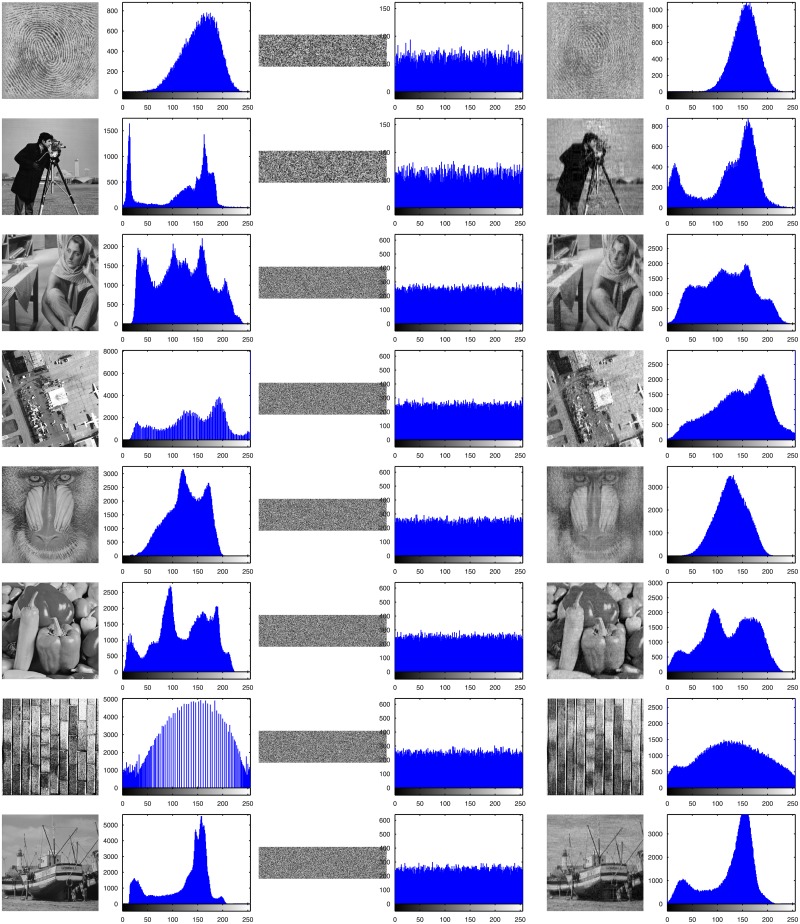
Histograms of the plain images, their corresponding encrypted images and recovered images.

The plain images, encrypted images and recovered images, and their corresponding histograms are shown in Fig 8. The histograms in the second column are from the plain images in the first column. These histograms have shapes that resemble some peaks or valleys, while all the histograms in the fourth column of encrypted images distribute very evenly and almost uniformly. The third column shows the encrypted images, of which the CR is 0.25 and the height is 1/4 of the original image. The recovered images are listed in the fifth column, while the corresponding histograms are listed in the sixth column. Because we use lossy SBL to compress the image with *CR* = 0.25, the recovered image in the fifth column is slightly blurred, and the histograms have lost some details with the peaks or valleys.

Nevertheless, these almost even histograms of encrypted images indicate that the QSBLA has a strong ability to resist histogram attacks. Moreover, the proposed approach can encrypt any images to flat and even histograms without exception. These results confirm that the QSBLA works very well for any types of image.

The results in this section verify that it is impossible to recover the plain image after encryption through the cryptosystem by analyzing its histogram and to make the statistical analysis attack successful when hackers try to attack the compressed and encrypted images with a very even histogram in the fourth row of Fig 8.

#### 4.5.2 Information entropy

Information entropy (IE) is the average rate at which information is produced by a stochastic source of data. Here, it is used to reflect the complexity or orderliness of the encryption system. The intensity of an 8-bit grayscale image has 2^8^ possible values ([0, 255]). The IE is defined as Eq (18) [70]:

$$\begin{array}{c} \text{IE}\left(Q_{i}\right)=-\sum_{i=0}^{255}p\left(Q_{i}\right)log_{2}\;p\left(Q_{i}\right), \end{array}$$ (18)

where *p*(*Q*_*i*_) is the probability that the pixel gray value *Q*_*i*_ exists in an image [63]. The maximum of IE is 8 when *Q*_*i*_ of an encrypted image has the same probability, i.e., $\frac{1}{256}$. The perfect uniform distribution of the encrypted image has the maximal IE, 8.

The IEs of plain images and corresponding compressed and encrypted images produced by different encryption schemes are listed in Table 4. The third column under QSBLA shows the entropies of the final encrypted compression images. It can be seen from this table that the IEs of the plain images are far below 8, while those of compressed and encrypted images are very close to the theoretical optimal value of 8. The IEs of QSBLA are within [7.9878, 7.9977]. Although the QSBLA achieves none of the 8 optimal values while both CHC and IC-BSIF obtain the optimal values 4 times, the IE range of [7.9878, 7.9977] by the QSBLA is very close to the theoretical optimal value, 8. It can be seen that, as a compression and encryption approach, the QSBLA is very comparable to the state-of-the-art encryption models regarding IE.

**Table 4:** The IE of the testing images.

Image	Input Images	Cipher images				
		QSBLA	HC-DNA [63]	CDCP [54]	CHC [55]	IC-BSIF [64]
Finger	7.1075	7.9880	7.9964	7.9969	7.9970	**7.9974**
Cameraman	7.1048	7.9878	7.9964	7.9976	7.9972	**7.9977**
Barbara	7.6321	7.9974	7.9993	7.9992	7.9992	**7.9993**
Airfield	7.1206	7.9974	7.9992	7.9992	7.9992	**7.9994**
Baboon	7.1391	7.9973	7.9992	7.9993	**7.9994**	7.9993
Peppers	7.5925	7.9977	7.9992	7.9993	**7.9994**	7.9992
Texture	6.5803	7.9971	7.9984	7.9993	**7.9994**	7.9993
Boats	7.0333	7.9973	7.9987	7.9993	**7.9994**	7.9993

We further use Peppers to compare the QSBLA with a compression and encryption scheme, Ref. [71], and the results are listed in Table 5, where *LCR* and *HCR* are the compression ratio of the low-frequency component and the high-frequency component respectively. The IEs of Peppers by QSBLA are much higher than those by Ref. [71]. All the IEs of Pepper under different CRs is greater than 7.997 and the maximum of Ref. [71] is only 5.5981. Although the comparison is under different *CR*, *LCR* and *HCR*, their encrypted images of QSBLA and Ref. [71] are still comparable for entropy performance. The possible reason is that the QSBLA conducts compression and then encryption while Ref. [71] performs encryption and then compression, and the latter will discard some less important information (zeros and values close to zero) in compression, losing the diversity of the information and decreasing the IE values.

**Table 5:** The IE compared with Ref. [71].

Image	CR	QSBLA	HCR, LCR	Ref. [71]
Peppers	CR = 0.25	7.9971	HCR = 0.2, LCR = 0.8	5.5981
	CR = 0.45	7.9985	HCR = 0.4, LCR = 0.8	4.8076
	CR = 0.50	7.9985	HCR = 0.2, LCR = 0.6	5.5914
	CR = 0.55	7.9986		
	CR = 0.65	7.9990		
	CR = 0.75	7.9991		

#### 4.5.3 Correlation analysis

The grayscale levels of two neighboring pixels in a natural image are always similar and thus are highly correlated. The correlation of two neighboring pixels in a natural image is usually close to 1. A good image encryption algorithm should produce an encrypted image with very low correlation to make sure it is impossible to deduce information from its neighbors.

The correlation coefficient *γ* is the most-widely used index to quantify the correlation, which can be formulated as Eq (19) [72].

$$\begin{array}{c} \begin{array}{cc} E(x) & =\frac{1}{N}\sum_{i=1}^{N}x_{i},\\ D(x) & =\frac{1}{N}\sum_{i=1}^{N}{\left(x_{i}-E\left(x\right)\right)}^{2},\\ cov(x,y) & =\frac{1}{N}\sum_{i=1}^{N}\left(x_{i}-E\left(x\right)\right)\left(y_{i}-E\left(y\right)\right),\\ \gamma & =\frac{cov(x,y)}{\sqrt{D(x)D(y)}}, \end{array} \end{array}$$ (19)

where *x* and *y* are the grayscale values of two neighboring pixels among the total *N* pixels in an image.

As shown in Table 6, we compute the correlation coefficients *γ* for all plain images and compressed and encrypted images at three directions, i.e., horizontal *γ*_*h*_, vertical *γ*_*v*_, and diagonal *γ*_*d*_ [63]. This table shows that the correlation coefficients of all the plain images are close to 1 in all directions, meaning high correlation, whereas those of all the compressed and encrypted images are slightly greater than 0, showing very low correlation. This result indicates that the QSBLA is able to effectively reduce the correlation to a very low degree, even with compression. The QSBLA outperforms the rest of the schemes on 3 out of 24 correlation coefficients, however, the other algorithms, HC-DNA, CDCP, CHC and IC-BSIF, achieve the optimal value 5, 3, 6 and 7 times. By comparing the results, it is obvious that the QSBLA has the same good performance in terms of correlation of the compressed and encrypted images.

**Table 6:** The correlation coefficients *γ* of the testing images.

Image	*γ*	Input images	Cipher images				
			QSBLA	HC-DNA [63]	CDCP [54]	CHC [55]	IC-BSIF [64]
Finger	*γ*_*h*_	0.5562	0.0013	0.0056	0.0006	**-0.0002**	0.0022
	*γ*_*v*_	0.6138	0.0111	-0.0021	-0.0059	-0.0031	**0.0021**
	*γ*_*d*_	0.4541	0.0125	0.0049	0.0033	0.0053	**-0.0024**
	*γ*_*h*_	0.9329	-0.0028	0.0076	-0.0022	-0.0069	**-0.0008**
Cameraman	*γ*_*v*_	0.9566	-0.0065	-0.0091	-0.0054	-0.0044	**-0.0032**
	*γ*_*d*_	0.9117	0.0018	-0.0012	0.0048	**0.0010**	-0.0020
	*γ*_*h*_	0.8940	-0.0013	0.0010	-0.0026	**0.0001**	-0.0017
Barbara	*γ*_*v*_	0.9572	-0.0006	**0.0004**	0.0006	0.0033	0.0022
	*γ*_*d*_	0.8942	0.0008	-0.0009	**0.0005**	-0.0014	-0.0013
	*γ*_*h*_	0.9375	0.0034	**-0.0004**	0.0010	0.0017	-0.0006
Airfield	*γ*_*v*_	0.9398	0.0007	**0.0002**	-0.0033	-0.0003	0.0018
	*γ*_*d*_	0.9068	-0.0027	-0.0026	0.0013	-0.0008	**-0.0005**
	*γ*_*h*_	0.8652	0.0074	0.0050	-0.0021	**0.0019**	0.0046
Baboon	*γ*_*v*_	0.7524	-0.0012	0.0030	**-0.0001**	0.0017	-0.0002
	*γ*_*d*_	0.7210	0.0011	0.0010	-0.0027	-0.0008	**0.0002**
	*γ*_*h*_	0.9733	**0.0007**	0.0009	-0.0015	-0.0017	-0.0008
Peppers	*γ*_*v*_	0.9763	0.0042	0.0041	-0.0012	**-0.0003**	-0.0026
	*γ*_*d*_	0.9650	-0.0012	0.0008	0.0017	**-0.0006**	-0.0011
	*γ*_*h*_	0.7532	0.0003	**0.0000**	-0.0022	-0.0009	0.0015
Texture	*γ*_*v*_	0.8491	**-0.0003**	0.0013	-0.0032	-0.0005	-0.0033
	*γ*_*d*_	0.7114	-0.0017	-0.0010	**-0.0003**	-0.0004	0.0033
	*γ*_*h*_	0.9631	0.0042	**0.0010**	-0.0010	0.0019	0.0021
Boats	*γ*_*v*_	0.9824	-0.0018	0.0010	-0.0007	0.0005	**0.0004**
	*γ*_*d*_	0.9527	**0.0002**	0.0002	0.0010	-0.0004	-0.0025

**Fig 9:**
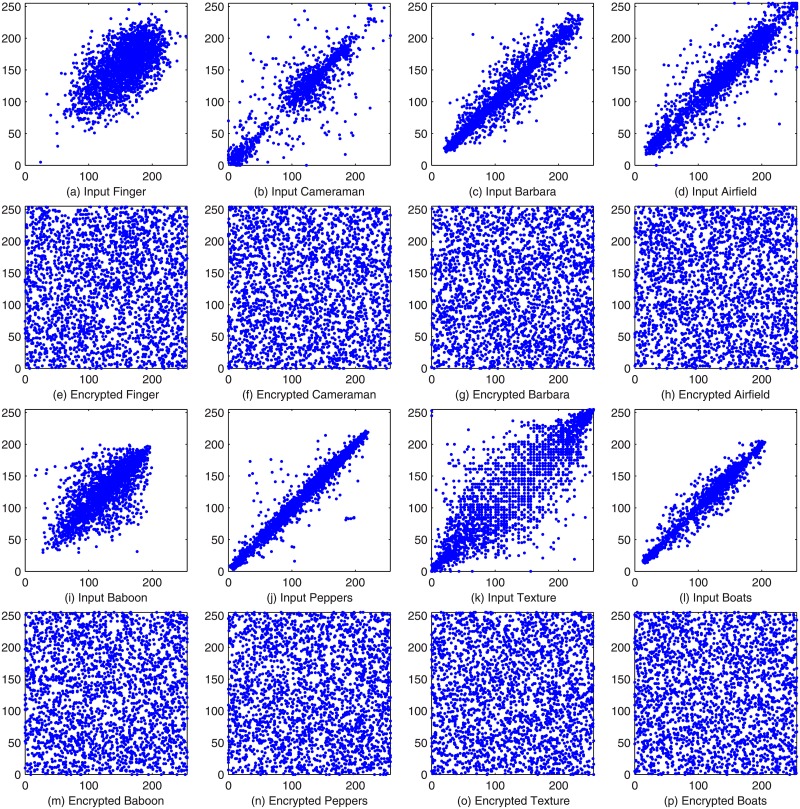
The adjacent-pixel distribution maps of the input images and the corresponding encrypted compression images in the horizontal direction.

To conduct a correlation analysis further, we also select 2500 pairs of neighboring pixels randomly in the horizontal direction from the plain images and the corresponding compressed and encrypted images by the QSBLA to show their distribution maps of neighboring pixels in Fig 9. The correlation values of plain images distribute near the diagonal area of a coordinate plane, showing strong correlation of the input plain images. In particular, the more the correlation points are close to the diagonal line that neighboring pixel distribution maps have, the higher the correlation of an image. For example, for plain images Peppers and Boats, their maps in Fig 9(j) and 9(l) show that these two images have higher correlation than the other 6 images. However, the gray values of compressed and encrypted images distribute randomly and evenly on the whole plane, showing very weak correlation of the compressed and encrypted images. The experimental results demonstrates that most of the correlation is eliminated by the QSBLA.

### 4.6 Analysis of resisting differential attacks

Based on the theory of cryptography, differential attack should be well defended by any image encryption schemes. Hence, any trivial change like a bit or a pixel change in a plain image should result in a totally different encrypted image.

For differential attack analysis, the number of pixels change rate (NPCR) and the unified average changing intensity (UACI) are two widely used performance indexes. The NPCR stands for the variation ratio of two encrypted images when their plain images are slightly changed on one bit or one pixel. The UACI defines the average intensity of the differences between the encrypted images from the plain image and the one bit or one pixel changed plain images. Theoretically, NPCR and UACI between two encrypted images, *Q*^1^ and *Q*^2^, can be defined as Eqs (20) and (21), respectively [73].

$$\begin{array}{c} \text{NPCR}=\frac{1}{wh}\sum_{i=1}^{h}\sum_{j=1}^{w}d_{ij}\times 100\%, \end{array}$$ (20)

$$\begin{array}{c} \text{UACI}=\frac{1}{wh}\sum_{i=1}^{h}\sum_{j=1}^{w}\frac{\left|Q_{ij}^{1}-Q_{ij}^{2}\right|}{255}\times 100\%, \end{array}$$ (21)

where *w* and *h* are still the width and the height of the image, and *d*_*ij*_ is defined as Eq (22):

$$\begin{array}{c} d_{ij}=\{\begin{array}{ccc} 0, & & Q_{ij}^{1}=Q_{ij}^{2},\\ 1, & & Q_{ij}^{1}\neq Q_{ij}^{2}. \end{array} \end{array}$$ (22)

The NPCR focuses on the number of pixels changing their values in the differential attack. The UACI concentrates on the average difference between the correct encrypted image and the encrypted image from differential attack. The expectations of NPCR and UACI of an encrypted image with 256 grayscale levels are 99.6094% and 33.4635% [73]. Generally, the more that NPCR becomes close to 100% and the larger that UACI is, the more effectively the encryption scheme can resist differential attacks.

We randomly choose one pixel in the plain images and only change one bit of the chosen gray value to compute the NPCR and UACI for one time. This process is repeated 10 times. And then the averaged NPCR and UACI of QSBLA, HC-DNA, CDCP, CHC and IC-BSIF are reported in Tables 7 and 8 as the final results.

**Table 7:** The average NPCR (%) of running the schemes 10 times.

Image	QSBLA	HC-DNA [63]	CDCP [54]	CHC [55]	IC-BSIF [64]
Finger	**99.6985**	52.9581	97.5395	99.5847	99.6124
Cameraman	99.5929	44.0302	99.5425	**99.6126**	99.6033
Barbara	**99.6208**	35.5757	99.5206	99.6047	99.6063
Airfield	99.6164	58.0984	**99.6182**	99.6161	99.6048
Baboon	**99.6245**	39.6885	99.5498	99.6132	99.6077
Peppers	99.6126	67.1383	**99.6583**	99.6088	99.6101
Texture	99.6121	65.2708	**99.6674**	99.6096	99.6123
Boats	99.6121	49.2368	**99.6440**	99.6078	99.6053

**Table 8:** The average UACI (%) of running the schemes 10 times.

Image	QSBLA	HC-DNA [63]	CDCP [54]	CHC [55]	IC-BSIF [64]
Finger	33.3370	21.7492	33.4814	33.4734	**33.5066**
Cameraman	33.4427	17.5397	**33.5157**	33.4502	33.4865
Barbara	33.4140	10.3142	33.4286	33.4632	**33.4811**
Airfield	33.4823	26.5238	**33.5091**	33.4644	33.4492
Baboon	**33.5939**	13.7956	33.4307	33.4501	33.4741
Peppers	33.4477	21.0504	33.4748	**33.4817**	33.4673
Texture	33.4713	28.0900	33.4650	33.4870	**33.4873**
Boats	33.4023	16.1896	33.4641	33.4645	**33.4712**

We can see from Table 7 that regarding NPCR, the QSBLA outperforms HC-DNA, CHC and IC-BSIF, and it also achieves comparable results with CDCP. QSBLA, CDCP and CHC achieve 3, 4 and 1 optimal NPCR values respectively. In Table 8, regarding UACI, QSBLA has one of the best records compared with all the other schemes, whereas HC-DNA still has the poorest results in all cases and IC-BSIF has 4 of the best records. The values of NPCR and UACI indicate that the QSBLA is able to resist differential attacks very well. It is worth noting that the proposed scheme includes compression while the other 4 methods do nothing about image compression.

### 4.7 Robustness analysis

Noise and data loss are inevitable for images during storage and transmission. A good JICE scheme should be able to resist noise or data loss. Theoretically, the compressed image is more difficult to be robust because the compression image converges more information into a smaller storage space. Compared with the encrypted lossless compression algorithms, any noise or data loss in lossy encryption algorithms should have more effects on the recovered image and thus lead to less robustness.

**Fig 10:**
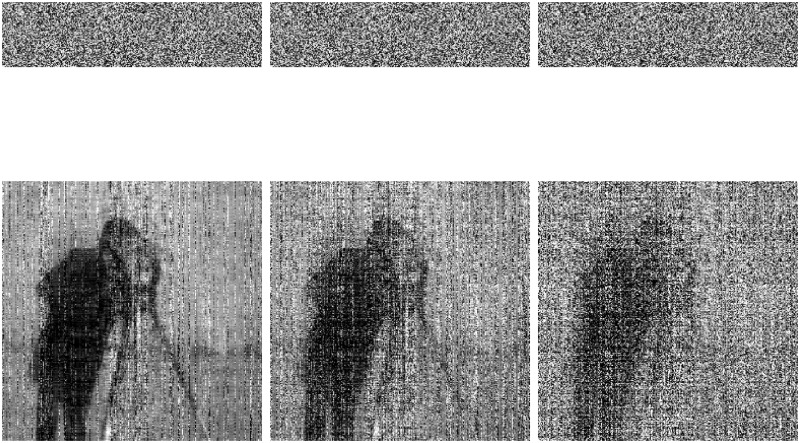
Robustness analysis results of noise. The compressed and encrypted Cameraman with 0.5%, 1% and 2% salt & pepper noise and its recovered image.

**Fig 11:**
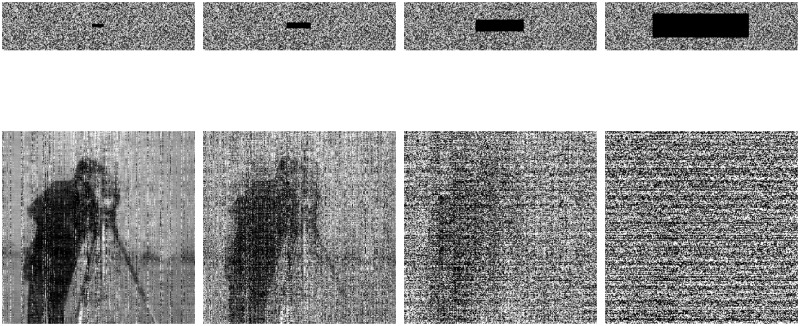
Robustness analysis results of data loss. The compressed and encrypted Cameraman with 0.4%, 1.56%, 6.25% and 25% data loss and its recovered image.

The results of robustness analysis by the QSBLA are shown in Figs 10 and 11. We first add 0.5%, 1% and 2% salt & pepper noise into the compressed and encrypted Cameraman with *CR* = 0.25, and the corresponding recovered images are shown in the first to the third column of Fig 10, respectively. It can be seen that, as far as 0.5% and 1% salt & pepper noise is concerned, although the recovered image contains much noise, it can recover the plain image to some extent. However, when the noise increases to 2%, we can only see some of the outline of Cameraman. When the encrypted compression image (*CR* = 0.25) has 0.4%, 1.56%, 6.25% and 25% data loss, the proposed QSBLA can recover Cameraman until data loss reaches 6.25% even though *CR* = 0.25 is a comparably high CR for CS, as shown in Fig 11. For 6.25% data loss, the decrypted image retains some information for us to recognize Cameraman, as shown in the third column of Fig 11. For 25% data loss, the main information of Cameraman about its contour is lost and the left information in the Fig 11 is random values. From the analysis, conclusively, the proposed QSBLA is robust to a certain extent.

### 4.8 Known-plaintext and chosen-plaintext attack analysis

**Fig 12:**
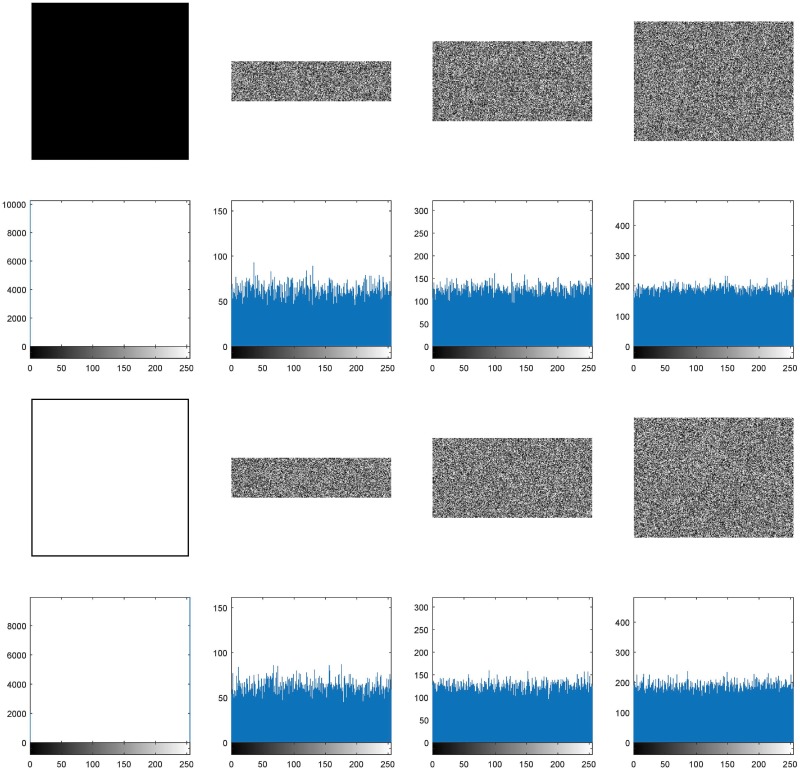
The compressed and encrypted images and their corresponding histograms with different *CR*s for all black and all white images. The first row is the plain all black image, and their corresponding compressed and encrypted images with *CR* = 0.25, 0.5 and 0.75, and the second row is the corresponding histograms of the images in the first row. The third and the fourth rows show the corresponding information of the all white image.

As analyzed previously, any tiny changes in the plain image can result in a totally different cipher image, so the proposed QSBLA can resist differential attacks, a typical chosen-plaintext attack. In addition, the security keys include y max and y min which are associated with the contents of the corresponding plain image. So different plain images will produce different security keys. Hackers usually use images of all black and all white to crack image encryption algorithms. The compressed and encrypted images of all black and all white with a size of 256 × 256 with different CRs are shown in Fig 12. It can be seen that all the compressed and encrypted images are all noise-like and all the corresponding histograms are very close to uniform distributions, showing that the proposed QSBLA has good encryption effect for both images of all black and all white. From the above analysis, we can see that the QSBLA is capable of resisting known-plaintext and chosen-plaintext attacks.

### 4.9 Computing time analysis

Different JICE algorithms have different performance on computing complexity. So the encryption and decryption time of different size images at different CRs is tested, and their results are listed in Tables 9 and 10. As we can see from the tables, like other JICE algorithms, generally speaking, the smaller CR or the smaller image size, the less computational time is. But there is an exception for *CR* = 0.75 regarding decryption, the decryption time drop significantly especially for images Cameraman, Barbara, Peppers. The reason is that SBL converges to the final solution in a shorter time compared with *CR* = 0.65 although it needs to compute more variables when *CR* = 0.75.

**Table 9:** The encryption time of different images under different compression ratios.

Image	Finger	Cameraman	Barbara	Airfield	Baboon	Peppers	Texture	Boats
CR = 0.25	0.8803	0.8544	3.4274	3.4145	3.3504	3.3952	3.3551	3.3578
CR = 0.45	1.4915	1.5186	5.9977	6.0576	6.0357	5.9701	6.0885	6.0384
CR = 0.50	1.6467	1.6760	6.7649	6.6411	6.6930	6.7148	6.7209	6.6202
CR = 0.55	1.8572	1.8419	7.5312	7.6224	7.4462	7.4166	7.3337	7.4377
CR = 0.65	2.1755	2.1954	8.7269	8.6258	8.7627	8.6462	8.7135	8.7965
CR = 0.75	2.5370	2.4888	9.9600	9.9105	9.8883	9.9209	9.9265	10.0201

**Table 10:** The decryption time of different images under different compression ratios.

Image	Finger	Cameraman	Barbara	Airfield	Baboon	Peppers	Texture	Boats
CR = 0.25	3.2699	3.3078	13.2795	13.7760	14.1945	13.6077	14.1224	12.8280
CR = 0.45	7.2216	7.3246	30.0707	30.0453	30.3387	30.6608	30.4666	29.8126
CR = 0.50	5.8985	7.8638	37.5421	38.3185	38.7335	38.5830	24.4251	36.6059
CR = 0.55	9.2485	9.4215	41.5784	42.1241	23.2554	41.6836	14.7176	41.5421
CR = 0.65	3.4091	11.8130	53.3911	18.4296	15.8421	26.8692	15.1310	52.7939
CR = 0.75	3.7706	3.9803	23.5817	15.7687	13.8143	17.2203	14.4416	54.9036

The comparison between QSBLA and Ref. [32] is listed in Tables 11 and 12. When *CR* = 0.75, the decryption time of QSBLA is less for Peppers. In other cases, the time is longer than Ref. [32]. We further compare the compression and encryption time with *CR* = 0.55 for Cameraman and Peppers, and the results are shown in Table 13. It can be seen that outperforms Ref. [39] but underperforms the other compared schemes. The computing time is possible improved by using parallel computing to optimize SBL and run it on GPU. This is our future work.

**Table 11:** The encryption time compared with Ref. [32].

Image	CR	QSBLA	Ref. [32]
Finger	CR = 0.25	0.8803	0.4536
	CR = 0.50	1.6467	0.4605
	CR = 0.75	2.5370	0.4545
Peppers	CR = 0.25	3.3952	0.9934
	CR = 0.50	6.7148	0.9925
	CR = 0.75	9.9209	1.0085

**Table 12:** The decryption time compared with Ref. [32].

Image	CR	QSBLA	Ref. [32]
Finger	CR = 0.25	3.2699	1.1374
	CR = 0.50	5.8985	1.1374
	CR = 0.75	3.7706	2.8476
Peppers	CR = 0.25	13.6077	5.0698
	CR = 0.50	38.5830	13.4131
	CR = 0.75	17.2203	22.0483

**Table 13:** The encryption time of different algorithms with *CR* = 0.55.

Image	QSBLA	Ref. [32]	Ref. [33]	Ref. [39]	Ref. [40]
Cameraman	1.8419	0.7134	0.3085	5.5668	0.4980
Peppers	7.4166	0.9988	0.5368	8.9744	0.9382

## 5 Conclusions

Image compression and image encryption are two important tasks in image processing. In this paper, we propose a novel approach that integrates a quantum chaotic system, sparse Bayesian learning and a 3D Arnold cat map, namely, QSBLA, for joint image compression and encryption. The novelty of the QSBLA is introducing SBL to compress images and using a 3D Arnold cat map to permute bit-level cubes. The extensive experiments demonstrate that the QSBLA has the ability to achieve good compression performance and is capable of resisting several types of attacks, showing that the QSBLA is promising for joint image compression and encryption. In the future, we will study the permutation on DNA-level data and extend the proposed QSBLA to joint image compression and encryption for color images.

## Supporting information

S1 File Data. All the data for the experiments in the paper.
(ZIP)
