# Supplementary figures and images for: Joint image compression and encryption based on sparse Bayesian learning and bit-level 3D Arnold cat maps
Source: PLoS One. 2019 Nov 18;14(11):e0224382. doi: 10.1371/journal.pone.0224382 (PMC6860426; doi:10.1371/journal.pone.0224382)

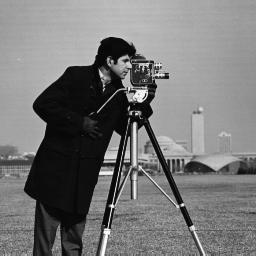

Supplement: S1 File — All the data for the experiments in the paper. (ZIP) [file pone.0224382.s001.zip › Cameraman.jpg]

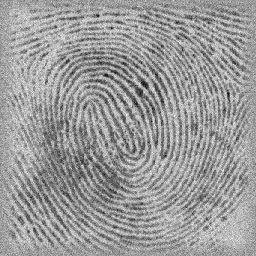

Supplement: S1 File — All the data for the experiments in the paper. (ZIP) [file pone.0224382.s001.zip › Finger.jpg]

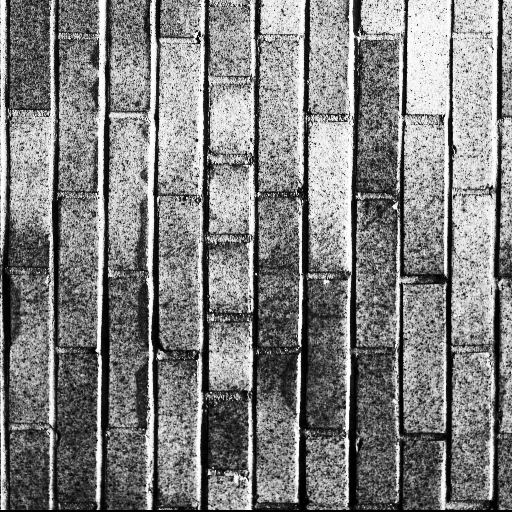

Supplement: S1 File — All the data for the experiments in the paper. (ZIP) [file pone.0224382.s001.zip › Texture.tiff]

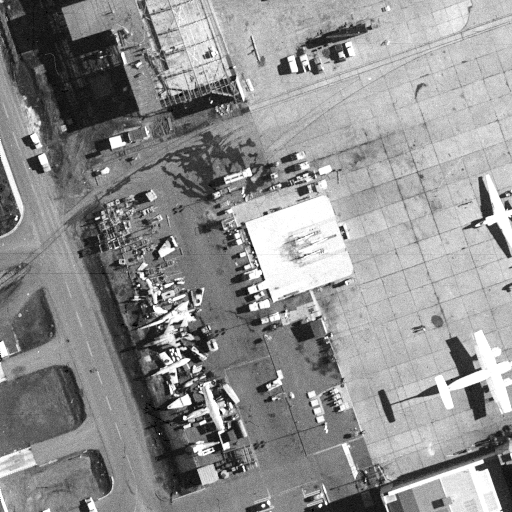

Supplement: S1 File — All the data for the experiments in the paper. (ZIP) [file pone.0224382.s001.zip › Airfield.tif]

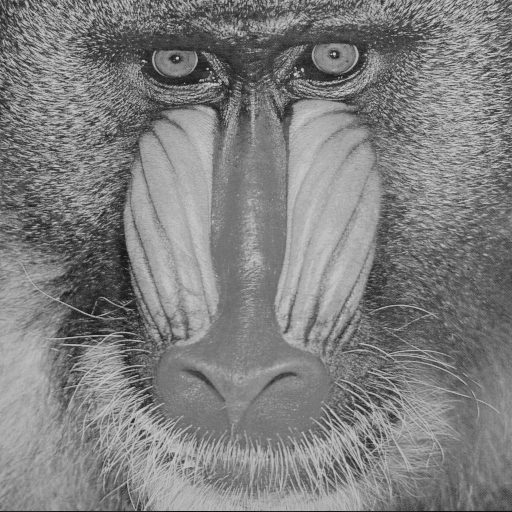

Supplement: S1 File — All the data for the experiments in the paper. (ZIP) [file pone.0224382.s001.zip › Baboon.bmp]

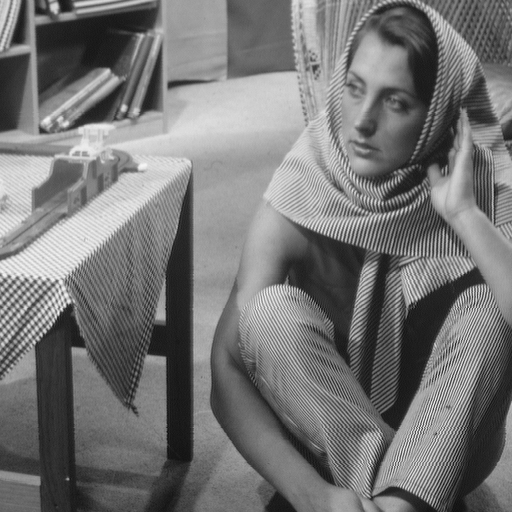

Supplement: S1 File — All the data for the experiments in the paper. (ZIP) [file pone.0224382.s001.zip › Barbara.bmp]

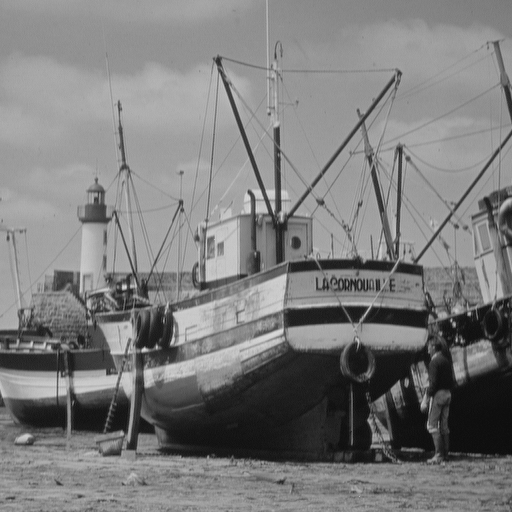

Supplement: S1 File — All the data for the experiments in the paper. (ZIP) [file pone.0224382.s001.zip › Boats.tif]
